# Acinetobactin-Mediated Inhibition of Commensal Bacteria by Acinetobacter baumannii

**DOI:** 10.1128/msphere.00016-22

**Published:** 2022-02-09

**Authors:** Gregory A. Knauf, Matthew J. Powers, Carmen M. Herrera, M. Stephen Trent, Bryan W. Davies

**Affiliations:** a Department of Molecular Biosciences, University of Texas at Austin, Austin, Texas, USA; b Center for Systems and Synthetic Biology, John Ring LaMontagne Center for Infectious Diseases, University of Texas at Austin, Austin, Texas, USA; c Department of Microbiology, College of Arts and Sciences, University of Georgia, Athens, Georgia, USA; d Department of Infectious Diseases, University of Georgia, Athens, Georgia, USA; University of Iowa

**Keywords:** *Acinetobacter*, acinetobactin, iron utilization

## Abstract

Acinetobacter baumannii is an important hospital-associated pathogen that causes antibiotic resistant infections and reoccurring hospital outbreaks. A. baumannii’s ability to asymptomatically colonize patients is a risk factor for infection and exacerbates its spread. However, there is little information describing the mechanisms it employs to colonize patients. A. baumannii often colonizes the upper respiratory tract and skin. Antibiotic use is a risk factor for colonization and infection suggesting that A. baumannii likely competes with commensal bacteria to establish a niche. To begin to investigate this possibility, we cocultured A. baumannii and commensal bacteria of the upper respiratory tract and skin. In conditions that mimic iron starvation experienced in the host, we observed that A. baumannii inhibits Staphylococcus epidermidis, Staphylococcus hominis, Staphylococcus haemolyticus and Corynebacterium striatum. Then using an ordered transposon library screen we identified the A. baumannii siderophore acinetobactin as the causative agent of the inhibition phenotype. Using mass spectrometry, we show that acinetobactin is released from A. baumannii under our coculture conditions and that purified acinetobactin can inhibit C. striatum and S. hominis. Together our data suggest that acinetobactin may provide a competitive advantage for A. baumannii over some respiratory track and skin commensal bacteria and possibly support its ability to colonize patients.

**IMPORTANCE** The ability of Acinetobacter baumannii to asymptomatically colonize patients is a risk factor for infection and exacerbates its clinical spread. However, there is minimal information describing how A. baumannii asymptomatically colonizes patients. Here we provide evidence that A. baumannii can inhibit the growth of many skin and upper respiratory commensal bacteria through iron competition and identify acinetobactin as the molecule supporting its nutritional advantage. Outcompeting endogenous commensals through iron competition may support the ability of A. baumannii to colonize and spread among patients.

## INTRODUCTION

The bacterial pathogen, Acinetobacter baumannii, is a cause of hospital-associated bacterial disease (1, 2). A. baumannii quickly acquires antibiotic resistance, which has led to an increased proportion of multi-drug resistant infections compared with other ESKAPE (Enterococcus faecium, Staphylococcus aureus, Klebsiella pneumoniae, Acinetobacter baumannii, Pseudomonas aeruginosa, and Enterobacter species) pathogens and entry to the CDC urgent threats list for bacteria most in need of new antibiotics (1, 3). Unfortunately, antibiotic development has stagnated (3). This has placed increased pressure on infection prevention to control A. baumannii. Environmental contamination (4) and asymptomatic patient colonization are important reservoirs for A. baumannii infection in clinical settings (4–20). Additionally, A. baumannii colonization is a major risk factor for future symptomatic A. baumannii infections, such as bacteremia and pneumonia, in individual patients (4, 6, 9, 10, 13, 15). Unfortunately, there is a dearth of knowledge relating to the mechanisms by which A. baumannii successfully colonizes patients.

In clinical settings, A. baumannii is commonly isolated from patient skin at various exposed sites as well as the upper respiratory tract (14–20). Recent antibiotic exposure is an important risk factor for general A. baumannii patient colonization (4, 9–11, 18). This suggests that A. baumannii competes with the host microbiota to colonize humans. Access to limited nutrients is often a focal point of microbial competition (21–23). A persistently limited resource in the human host is iron. This is due to the human host sequestering iron from bacteria in addition to microbial use of this finite resource (21–23). Under iron limited conditions it has been reported that pathogenic and commensal bacteria can be induced to exhibit competitive behaviors. For example, Staphylococcus lugdunensis can inhibit Staphylococcus aureus growth under iron limiting conditions via the secretion of the cyclic peptide lugdunin (24, 25) and Escherichia coli Nissile decreases Salmonella enterica Typhimurium colonization *in vivo* via competition for iron (26).

Here we investigated the ability of A. baumannii to compete with commensal bacteria common to the skin and upper respiratory track. We discover that under iron limiting conditions, A. baumannii inhibits the growth of common microbiome skin inhabitants Corynebacterium striatum, Staphylococcus epidermidis, Staphylococcus hominis and Staphylococcus haemolyticus. Through genetic and biochemical studies, we demonstrate that the A. baumannii siderophore acinetobactin is an important factor in this competition. Cumulatively, these data suggest a role for acinetobactin in A. baumannii host colonization and commensal competition.

## RESULTS

### A. baumannii inhibits the growth of commensal bacteria under iron limiting conditions.

**FIG 1:**
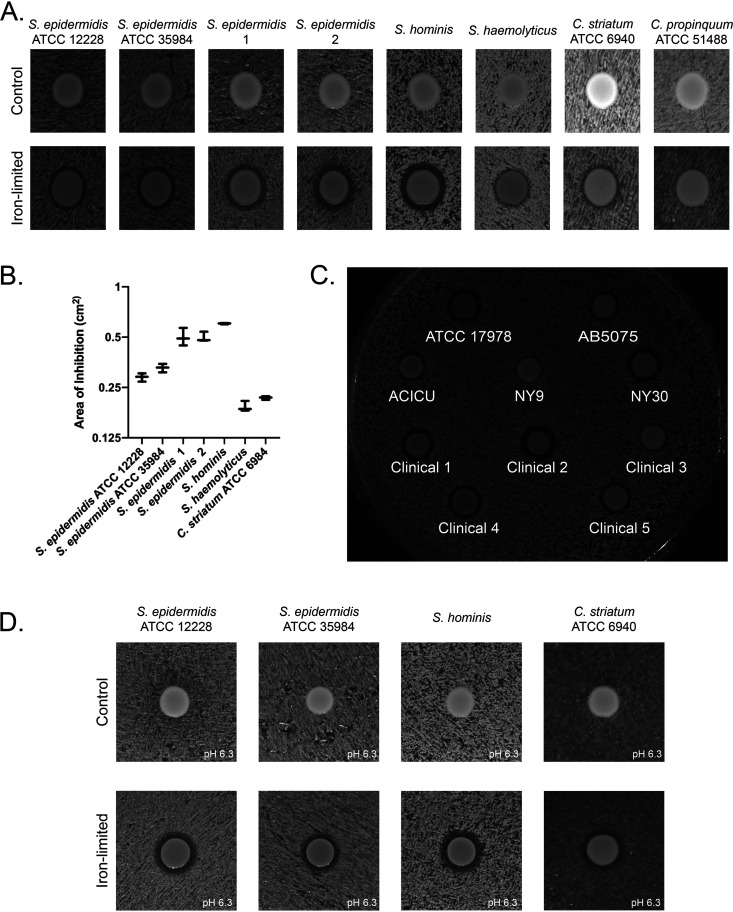
A. baumannii inhibits growth of Staphylococcus and *Corynebacterium* species under iron-limited conditions. (A) A. baumannii 17978 was spotted on a lawn of the indicated Staphylococcus or *Corynebacterium* species, grown on nutrient rich agar alone (control) or with 200 μM the iron chelator 2,2’-dipyridyl (Iron limited). A. baumannii generated a zone of clearance against all strains except *C. propinquum* ATCC 51488 under iron limited conditions. Experiments were performed in triplicate. A representative image is shown. (B) Quantification of A. *n* = 3 per competition for inhibited bacteria. The error bars represent the standard error of the mean. (C) Multiple laboratory and clinical A. baumannii isolates inhibit the growth of S. hominis under iron depleted conditions. (D) A. baumannii 17978 inhibits representative Staphylococcus and *Corynebacterium* species at pH 6.3 (Buffered using 50 mM MES.) in addition to standard culture pH conditions.

The microbiome of human skin and upper respiratory tract is less diverse than that of the gastrointestinal tract. Staphylococcus spp. and *Corynebacterium* spp. are prominent commensal bacteria of the skin and entire upper respiratory tract that invading pathogens must compete with to establish colonization and or infection (21, 22, 27–32). The localization of these bacteria at these sites suggests the possibility of competition with A. baumannii. To begin we selected representative bacteria documented in ATCC (S. epidermidis 12228, S. epidermidis 35984, C. striatum 6940 and *C. propinquum* 51488) and 4 bacterial isolates from the skin and nasal cavity of a healthy human volunteer (two S. epidermidis isolates, S. hominis and *S. haemolyticus*). We hypothesized that A. baumannii may influence the growth of commensal bacteria to promote its own colonization. To test this hypothesis *in vitro* we initially co-plated A. baumannii strain 17978 with strains of S. epidermidis, S. hominis, *S*. *haemolyticus*, C. striatum and *C*. *propinquum* on nutrient rich medium, but did not observe any effect of A. baumannii on the growth of these bacteria (Fig. 1A).

Compared with laboratory medium, the skin and upper respiratory track are nutrient poor niches. Scarce resources have been shown to trigger changes in microbial community dynamics, such as through effector molecules or altered growth through preferential resource acquisition (21–23). Iron is a scarce nutrient that bacteria have been shown to compete for in many niches (21, 23–25, 33). Therefore, we hypothesized that competition between A. baumannii and commensal bacteria may also be induced under iron limiting conditions. Iron limitation can be mimicked *in vitro* through the use of the iron chelator 2,2’-dipyridyl. To test this possibility, we repeated co-plating A. baumannii strain 17978 with the same set of commensal bacteria. Under iron depleted conditions we observed that A. baumannii 17978 inhibited the growth of all of the Staphylococcus species and C. striatum but, interestingly not *C. propinquum* (Fig. 1A). Furthermore, the strains of Staphylococcus species and C. striatum had different sensitivities to A. baumannii inhibition suggesting variable resistance to A. baumannii competition at the strain level (Fig. 1B). To examine the distribution of this phenotype among A. baumannii isolates we tested several clinical and laboratory A. baumannii strains for the ability to create a zone of inhibition against S. hominis. Each A. baumannii strain produced a zone of clearance of varying size against S. hominis indicating that this phenotype is widespread among A. baumannii strains (Fig. 1C).

The pH of the skin and the upper respiratory tract can vary from ∼5 to 7 (29, 34, 35). It is known that pH can play an important role in bacterial interactions and growth (36, 37). This suggests that an *in vivo* relevant pH change may affect the A. baumannii inhibitory phenotype since, standard tryptic soy and brain and heart infusion agar is about pH 7.3–7.4. To determine if pH influences competition, we repeated our assays using medium buffered at pH 6.3. Under these conditions we observed the same inhibitory phenotype for A. baumannii with regard to a set of representative S. epidermidis, S. hominis, and C. striatum strains (Fig. 1D).

### Acinetobactin biosynthesis influences A. baumannii commensal inhibition.

Zone of inhibition phenotypes regulated by iron concentration have been reported for both siderophores that acquire iron for the secreting organism (38) and secreted antimicrobials that kill competing bacteria (25, 32). To begin investigating genetic factors facilitating A. baumannii competition in an unbiased manner, we screened an ordered A. baumannii AB5075 transposon (T26) library (39) for reduction in the size of the zones of inhibition with S. epidermidis ATCC 12228 under iron limiting conditions. To increase the stringency of our analysis, we filtered the list of potential genes keeping only those for which three or more unique transposon insertions were identified as having a reduced zone of inhibition. This generated a list of 12 genes (Table 1). The genes listed in Table 1 is comprised solely of hits in which three or more different transposon insertion alleles of the same gene were identified in the screen as having a reduced zone of inhibition. Genes encoding the iron siderophore acinetobactin biosynthesis and transport operon represented half of the genes (6/12) in the filtered list. The remaining genes encoded functions associated with purine metabolism, biotin biosynthesis, gene regulation (putative RpoE homologue (40)), and an unknown function. The importance of iron chelation to the inhibitory phenotype A. baumannii produces suggests that acinetobactin is a strong candidate for playing an active role in the observed inhibition of Staphylococcus species and C. striatum.

**TABLE 1:** AB5075 Genes with Three or More Transposon Allele Disruptions Resulting in Loss of S. epidermidis Inhibition

Locus	Gene name	Putative function
ABUW_0251	–	GNAT domain-containing protein
ABUW_0988	–	putative RNA polymerase Sigma E (40)
ABUW_1169	*basA*	non-ribosomal peptide synthetase
ABUW_1170	*basB*	non-ribosomal peptide synthetase
ABUW_1179	*basD*	nonribosomal peptide synthetase
ABUW_1180	*basE*	2,3-dihydroxybenzoate-AMP ligase
ABUW_1182	*basG*	histidine decarboxylase
ABUW_1176	*bauB*	ferric acinetobactin transport system periplasmic binding protein
ABUW_3120	*bioA*	adenosylmethionine-8-amino-7-oxononanoate transaminase
ABUW_3886	*purE*	phosphoribosylaminoimidazole carboxylase, catalytic subunit
ABUW_1532	*purH*	phosphoribosylaminoimidazolecarboxamide formyltransferase/IMP cyclohydrolase
ABUW_0981	*purM*	phosphoribosylformylglycinamidine cyclo-ligase

**FIG 2:**
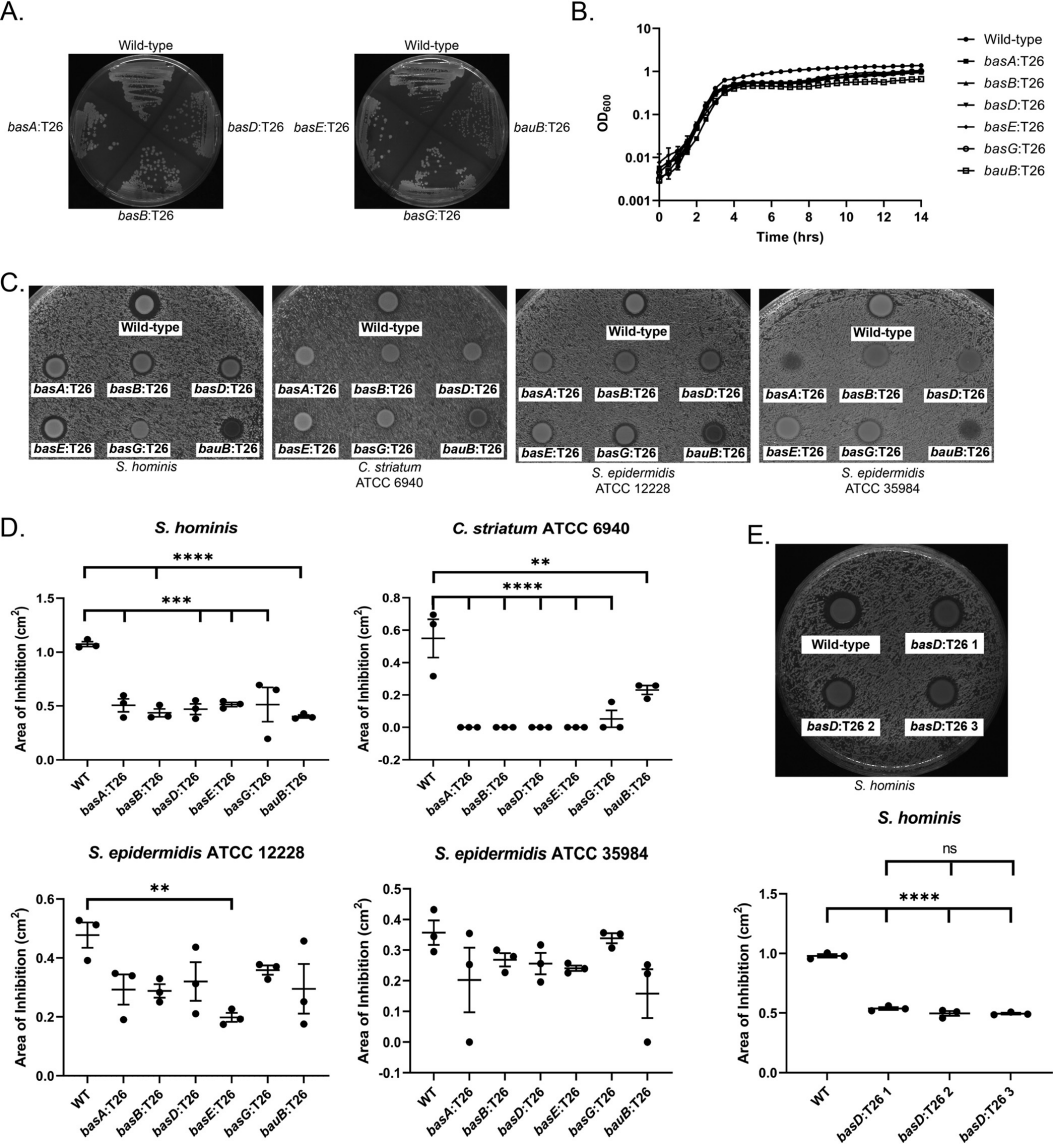
A. baumannii AB5075 Acinetobactin mutants exhibit decreased inhibition of multiple bacteria. (A) Growth of the parent and transposon mutant strains of A. baumannii AB5075 on 300 μM 2,2’-dipyridyl. (B) Growth curves of wild-type and transposon mutant strains of A. baumannii AB5075 grown in 300 μM 2,2’-dipyridyl tryptic soy broth. Growth curves were conducted in triplicate and plotted on a logarithmic scale. The error bars represent the standard error of the mean. (C) A. baumannii AB5075 transposon mutant and wild-type zones of inhibitions on iron limited media. Zone of inhibition assays were conducted in triplicate. (D) Quantification of the zones of inhibition in C. The error bars represent the standard error of the mean. An ANOVA with multiple comparisons, comparing each mutant to the control wild-type strain, was used to assess significance. (E) A. baumannii AB075 wild-type and three *basD*:T26 isogenic mutant zones of inhibition and quantification. The error bars represent the standard error of the mean. An ANOVA with multiple comparisons was used to assess significance. (⁕⁕, *P ≤ *0.01; ⁕⁕⁕, *P ≤ *0.001; ⁕⁕⁕⁕, *P ≤ *0.0001).

The acinetobactin biosynthesis pathway has been identified in the Acinetobacter genus as a virulence factor important for iron acquisition during infection (41–45). Additionally, acinetobactin is known to be upregulated in iron limiting conditions (46). Interestingly, despite A. baumannii AB5075 encoding genes to produce more than one siderophore (42), similar to other A. baumannii strains (46), only the acinetobactin siderophore biosynthesis pathway mutants showed decreased inhibition in our screen (Table 1). The loss of an inhibitor zone could be due to either a loss of the inhibitor compound, a reduced growth rate for the A. baumannii mutant or both. The growth of each acinetobactin transposon mutant on solid media, the condition used to examine the phenotype of interest, was similar to the parental strain (Fig. 2A). Additionally, the growth rates of the transposon mutants were comparable to the wild-type strain in liquid culture (Fig. 2B). This suggests that the other siderophore systems are sufficient to maintaining growth of AB5075 under our iron limited conditions. To verify the transposon library screen results, we compared wild-type A. baumannii AB5075, and transposon mutants *basA*:T26, *basB*:T26, *basD*:T26, *basE*:T26, *basG*:T26, and *bauB*:T26 inhibition of S. epidermidis ATCC 12228, ATCC 35984, S. hominis, and C. striatum ATCC 6940 on 2,2’-dipyridyl medium (Fig. 2C). Our results demonstrate that, compared with the wild type A. baumannii 5075 strain, each acinetobactin biosynthesis mutant showed a significant reduction in zone of inhibition against S. hominis and C. striatum (Fig. 2D). The mean zone of inhibition of each mutant against S. epidermidis ATCC 12228 was also smaller compared with the wild-type strain, but only the *basE* mutant showed a statistically significant reduction in zone size. Additionally, mutant zones of inhibition for S. epidermidis ATCC 35984 were generally lower than those for the wild-type strain, but none were statistically significant. These results highlight that the importance of acinetobactin for A. baumannii competition will likely vary with the competing bacteria. Some mutants (*basA*, *bauB*) also showed variations in growth when cocultured with different bacteria under iron limited conditions. While the reason for this is unclear and could be the result of numerous possible causes, this phenotype appeared in each replicate of the experiments. The residual zone of clearance around some of the transposon mutants observed with S. epidermidis (12228 and 35984) and S. hominis suggests A. baumannii AB5075 may have additional factors it employs for competition under iron limited conditions.

In sum, our genetic data suggests that acinetobactin plays an important role in interactions between A. baumannii and nasal and skin commensals. The structure and size of the acinetobactin biosynthesis operon negates simple complementation. However, identifying multiple Tn mutants in strain AB5075 all clustering in the acinetobactin biosynthesis pathway, and influencing inhibition of nasal and skin commensals (Fig. 2C), supports an important role for acinetobactin in A. baumannii competition. Furthermore, we tested three separate transposon insertion mutants of *basD* identified in the transposon library screen and found similar inhibition for the three isogenic mutants when tested in triplicate for zones of inhibition against S. hominis (Fig. 2E). To provide direct evidence for acinetobactin in commensal inhibition we proceeded to biochemical studies.

### A. baumannii produces acinetobactin during competition under iron-limited conditions.

Up to this point, our genetic results indicate that acinetobactin biosynthesis is important for the *in vitro* inhibition of Staphylococcal species and C. striatum. However, this does not demonstrate that acinetobactin is actually produced or present when commensal bacteria are inhibited. To address this, we first demonstrated that acinetobactin could be isolated from wild-type A. baumannii and second, we used MALDI-IMS (MALDI Imaging Mass Spec) to further investigate the production of acinetobactin and its correlation with inhibition of commensals using S. epidermidis as the test case.

**FIG 3:**
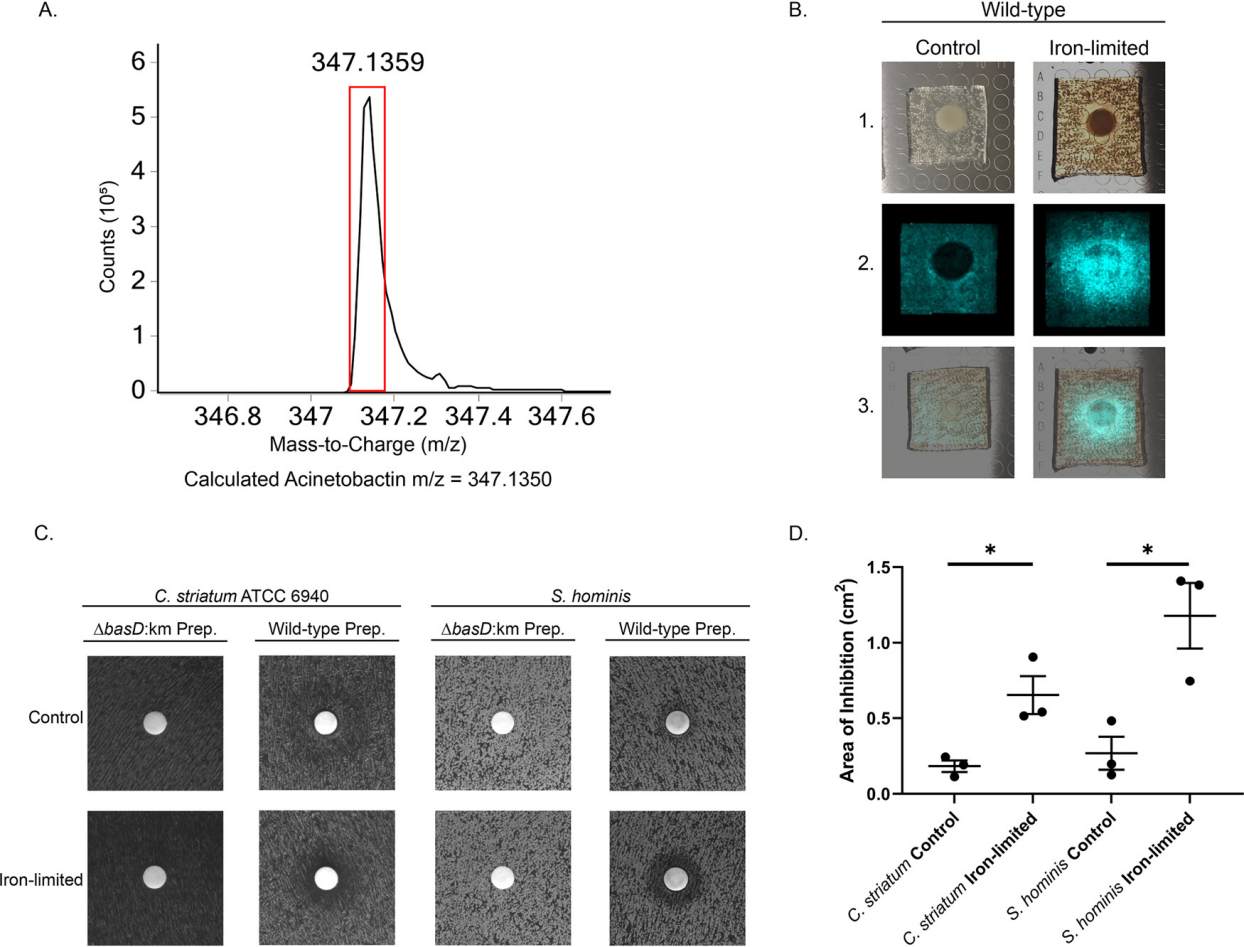
Biochemical evidence for acinetobactin-mediated inhibition. (A) Accurate mass spectra of the monoisotopic peak for acinetobactin preps purified from wild-type A. baumannii ATCC 17978. The red box represents where the peak is expected to be for acinetobactin. The theoretical *m/z* of monoisotopic acinetobactin is 347.1350, error 2.5 ppm and the observed *m/z* was 347.1359. (B) MALDI-IMS of acinetobactin production. 1. Images of wild-type 17978 spotted on a lawn of S. epidermidis 12228 on medium with (iron-limited) or without (control) 2,2’-dypyridyl. 2. Mass spectrometry analysis of the presence of acinetobactin. Presence of acinetobactin is false colored blue. 3. Overlap of 1 and 2. Experiments were repeated in triplicate and a representative image is shown. (C) HPLC prepped acinetobactin was added to a filter disk on a lawn of C. striatum or S. hominis on either 200 μM or 0 μM 2,2’-dypyridyl. A zone of inhibition indicates growth inhibition. Experiments were replicated and a representative image is shown. HPLC preps from a *basD*:km mutant was used as the control for inhibitory activity. (D) Quantification of the zones of inhibition for wild-type preps in C. The mean and standard error of the mean is shown in addition to the individual measurements. Significance was tested using a paired T-test comparing the control and iron-limited samples for each bacterial species. (⁕⁕, *P ≤ *0.05).

We began by isolating acinetobactin from wild-type A. baumannii ATCC 17978 as previously described (47). A. baumannii was grown for 48 h in 1 liter of M9 minimal media with succinate as the carbon source. Acinetobactin was isolated from the supernatant via XAD-7HP resin and preparatory HPLC followed by lyophilization to obtain the crude product mass. The lyophilized product was then rehydrated with sterile deionized water. The presence of acinetobactin was confirmed by mass spectrometry. The high-resolution spectra observed for monoisotopic acinetobactin in our preparation is shown in Fig. 3A. The observed *m/z* matches the theoretical *m/z* of acinetobactin. This demonstrates that our A. baumannii ATCC 17978 strain can produce acinetobactin and we successfully isolated a crude product containing acinetobactin.

MALDI-TOF mass spectrometry imaging allows for the determination of molecule production and location in two-dimensional space. We observed that only when grown in iron-limited conditions did the A. baumannii 17978 parental strain noticeably produce acinetobactin that diffuses into the S. epidermidis lawn (Fig. 3B). Importantly, acinetobactin appears to be dispersed throughout the zone of inhibition as would be expected for a secreted factor. This is an important finding since, if acinetobactin was capable of inhibiting some species of bacteria it should be present where the inhibition is taking place.

### Purified acinetobactin inhibits commensal bacteria growth.

The circumstantial evidence that acinetobactin can inhibit some species *in vitro* produced through the use of MALDI-TOF mass spectrometry is compelling. However, it is still indirect evidence. To directly determine whether acinetobactin can inhibit bacteria *in vitro*, we used purified acinetobactin and an identical preparation from a 17978 *basD*:km mutant culture. We selected theses strains as 17978 has a well-defined iron acquisition system including, acinetobactin, and is genetically tractable for producing whole gene deletions. We performed a disc diffusion assay of isolated acinetobactin and a *basD*:km control on S. hominis and C. striatum grown on iron-limited media and control media. For this assay, we plated the species described and applied a single dose of 500 mg of crude acinetobactin HPLC product, or a *basD*:km control preparation following the same protocol, to the diffusion disc before incubating overnight at 37°C. This assay showed that purified acinetobactin can form a zone of inhibition against C. striatum and S. hominis (Fig. 3C). These data demonstrate that acinetobactin can inhibit S. hominis and C. striatum in an iron-dependent manner. These zones of inhibition were quantified and show a significant difference in acinetobactin inhibition between the control and iron-limited conditions (Fig. 3D). These biochemical results further support our genetic analysis indicating that acinetobactin helps A. baumannii to inhibit the growth of nasal and skin commensals.

## DISCUSSION

Differences in siderophore production among closely related species might impact their susceptibility to A. baumannii inhibition in this study. For example, our data shows that *C. propinquum*, a species that likely inhibits coagulase negative staphylococcus through siderophore production (38), is resistant to A. baumannii inhibition compared with C. striatum. This suggests that the propensity of A. baumannii to establish itself in a microbiome may be variable between different host microbiomes in part as a function of iron acquisition capabilities. This is further suggested by the strain level variation in zones of inhibition depending on the S. epidermidis strain. This deserves further investigation to fully understand the role of iron competition in A. baumannii colonization.

Acinetobactin biosynthesis is a widely distributed capability of A. baumannii strains (42, 45, 46). Additionally, acinetobactin can bind iron in a wide range of pHs present in many different niches in the human body (47). Therefore, based off our results, we believe acinetobactin may play a major role in the success of A. baumannii in the human body in colonization in addition to its known importance in infection. While the A. baumannii AB5075 acinetobactin biosynthesis mutants exhibited decreased inhibition, the inhibition was not fully eliminated for all bacteria tested here. It is known that different A. baumannii strains are often capable of producing additional siderophores (42, 46). This suggests that in AB5075 the other siderophores may play a role in commensal inhibition despite our transposon library results only suggesting acinetobactin. To determine the ultimate extent of acinetobactin’s role in these processes other siderophores in A. baumannii’s arsenal should be explicitly investigated for a role in colonization and commensal competition.

While the *in vitro* inhibition experiments in this study focused on representative bacterial species of the upper respiratory and skin it is not clear how the same pairwise interactions would play out *in vivo*. Beyond these types of pairwise competitions, it is even less clear how the multi-domain web of interactions that take place *in vivo* may or may not impact A. baumannii colonization such as the metabolic capabilities, biofilm formation, resistance and/or manipulation of host defense factors and growth rates of A. baumannii strains and commensal microorganisms.

Through the experiments described here we have begun to investigate a possible role for acinetobactin in competition for iron in A. baumannii colonization. Given the importance of iron acquisition to survival in the microbiome, we expect that these experiments will be able to provide orientation for additional studies of host colonization more broadly. Finally, these data suggest a potential route to target A. baumannii to reduce the risk of A. baumannii spread and infection. This could potentially be done using antibiotic-siderophore conjugates as an approach that may prove fruitful in decolonization of patients in medium to long-term medical settings resulting in improved infection control. This study also suggests that commensal bacteria resistant to A. baumannii iron competition could offer an additional option for A. baumannii infection prevention or treatment.

## MATERIALS AND METHODS

### Isolation and Identification of Bacterial Species.

Bacterial isolates were isolated by swabbing the anterior nares, back and forehead of a healthy volunteer. These samples were then streaked on tripticase-soy agar and allowed to grow for 48 h at 30°C. Single colonies were then streaked and grown on new plates to ensure the isolation of individual species. Then frozen stocks were prepared for each selected isolate. Isolates were then subjected to 16S amplicon sequencing using the 27F-HT/1492R-HT primer pair (48) for PCR amplification.

### Zone of Inhibition Assays.

Zone of inhibition assays were carried out using Tripticase-soy media for Staphylococcus species and Brain and Heart Infusion media for Corynebacterium species 7.5 g of agar was added to 500 mL of growth media to form solid media. 2,2’-dipyridyl was added to media at the specified concentrations after being dissolved in dimethyl sulfoxide. 2,2’-dipyridyl was used at 200 μM for A. baumannii 17978 zones of inhibition, the transposon library screen, and when comparing the zones of inhibition formed by multiple A. baumannii isolates. Three hundred micometers of 2,2’-dipyridyl was used for assessing A. baumannii AB5075 transposon mutant zones of inhibition compared with the parental strain. Then 100 μL of the described bacteria at an OD_600_ ∼0.005, diluted in phosphate-buffered saline (PBS), was spread on the plate using glass beads to form a bacterial lawn. Then 10 μL of A. baumannii at an OD_600_ ∼1.0 was spotted on the plate and allowed to dry. Following, the spotting of A. baumannii the plate was incubated at 37°C overnight. When making dilutions of either A. baumannii or the species to be inhibited bacteria were grown overnight at 37 degrees on solid media then colonies were suspended to the correct optical density in PBS.

Where images are cropped to show a single zone of inhibition that zone of inhibition is the only zone of inhibition on a plate. When multiple zones of inhibition are performed on the same plate to compare inhibiting strains all strains tested are shown. Larger plates (150 × 22 mm) were used when more than four inhibiting strains were compared. In this circumstance the volume of inhibited bacteria was increased in proportion to the change in the size of the plate compared with plates used otherwise.

Measurements of the area of inhibition were performed in triplicate with a known scale for measurements included in each image. The measurements were performed using ImageJ. To acquire the area of the zone of inhibition, the area of only the A. baumannii spot was subtracted from that of the inhibition area and spot.

### Transposon Library Screen.

The transposon library was carried out on solid Tripticase-soy agar plates containing 200 μM 2,2’-dipyridyl. First a lawn of S. epidermidis ATCC 35984 was plated with 200 μL at 0.005 OD600. Then 96-well plates containing the ordered Three Allele A. baumannii AB5075 Transposon Library (39) were thawed and spotted onto the plates using a V&P 96-well replicater (VP 407, 1.5 μL transfer) and allowed to dry. This was carried out for all plates in the library. The plates were incubated overnight at 37°C. The following day colonies of A. baumannii AB5075 with a decreased or absence of a zone of inhibition were recorded and identified based on their position within a given 96-well plate. Select transposon mutants were then PCR verified and sanger sequenced.

### Genetic Manipulations and Fitness Assays.

Gene deletion and complementation was carried out as previously described (49) with single colony isolation carried out on tripticase-soy media.

For liquid growth curves A. baumannii strains were grown overnight at 37°C on tripticase-soy agar containing the appropriate antibiotic. Colonies were then suspended in tripticase-soy media to ∼0.005 OD_600_ before allocation into a 96-well plate in triplicate. A 14-h growth curve at 37°C with continuous shaking with reads every 15 min in a SpectraMax Plus 384 spectrometer. When plotting every half hour time point was plotted as an average with standard error of the mean error bars.

When streaking bacterial strains for growth assessment on tripticase-soy plates with 0 or 200 μM 2,2’-dipyridyl were prepared. Then, the plates were divided into quadrants and mutant and wild-type strains were streaked from frozen stocks from the outer edge toward the middle. Wild-type A. baumannii was streaked on each plate as a control.

### MALDI-IMS (MALDI Imaging Mass Spec).

Cultures of A. baumannii and S. epidermidis were normalized to an OD of 0.5 and 0.005 respectively. Microcolony lawns of S. epidermidis were spread on 10 mL LB plates with or without 100 μM 2,2’-dipyridyl. After plates were dried, 2.5 μL of the appropriate A. baumannii strain were spotted. Plates were incubated overnight. The following day, agar was excised and laid onto a coarse ground MALDI target plate (Bruker). The target plate was coated with a 25 mg/mL ATT matrix in 50% Acetonitrile + 0.1% TFA using a HTX TM-Sprayer at a flow rate of 0.2 mL/min. Samples were subsequently imaged by a MALDI Autoflex Speed (Bruker) in reflectron positive mode. Data were analyzed using flexImaging (Bruker) and images generated by false coloring based on spectral intensity.

### Purification of Acinetobactin.

Acinetobactin was purified as previously described (47). A. baumannii 17978 wild-type and *basD*:km strains were grown for 48 h in 1L of M9 minimal media with succinate as the carbon source. Then the supernatant was reserved following centrifugation and adjusted to pH 6.0 using concentrated citric acid. Following pH adjustment, 25g of Amberlite XAD-7 resin was added to the supernatant and placed on an orbital shaker for 4 h at 100 rpm and room temperature. Then the resin was filtered and washed 4 times with de ionized water methanol extraction using a total volume of 1.1 L. The methanol was rotovaped to concentrate the solution for HPLC use. A HPLC gradient of 5% to 95% solvent B over 45 min with, an acinetobactin formic acid salt elution time at ∼21 min. Samples were lyophilized to obtain product mass collected. Following rehydration with sterile deionized water Agilent Technologies 6546 Accurate-Mass Q-TOF LC/MS was used to verify the presence of acinetobactin in the collected fractions from wild-type cultures and the absence of acinetobactin in the collected fractions from *basD*:km mutant cultures.

### Purified Acinetobactin Zones of Inhibition.

Lyophilized HPLC preps from the same elution time point for wild-type and Δ*basD*:km strain cultures were suspended to the same volume using deionized water. C. striatum and S. hominis were streaked from frozen stocks and grown overnight at 37°C. Control (0 μM 2,2’-dipyridyl) and iron-limited (200 μM 2,2’-dipyridyl) media was prepared for each strain. Each bacterial strain was scraped from the overnight plate and diluted in PBS to an optical density of 0.005. Then 100 μL of each bacterial suspension was plated on each respective agar plate. The diffusion disc was added to the center of each plate and approximately 500 μg of wild-type crude product was added to the diffusion discs for the wild-type preps. An equivalent volume of Δ*basD*:km crude product prep as the wild-type prep was used as the ΔbasD:km prep to control for contaminants affecting bacterial growth. Images were quantified by subtracting the area of the disc from the area of the zones of inhibition using Image J.

### Strains, primers, and plasmids.

The strains, primers, and plasmids utilized are listed in Tables 2 and 3.

**TABLE 2:** Strains utilized

Strain name	Source
A. baumannii 17978 wild type	ATCC
A. baumannii 17978 *basD* deletion	This study
A. baumannii AB5075 wild type	Gallagher et al. (2015) (39)
A. baumannii AB5075 *bauB* transposon mutant, tnab1_kr121127p08q178	Gallagher et al. (2015) (39)
A. baumannii AB5075 *basA* transposon mutant, tnab1_kr121203p08q104	Gallagher et al. (2015) (39)
A. baumannii AB5075 *basG* transposon mutant, tnab1_kr130909p02q141	Gallagher et al. (2015) (39)
A. baumannii AB5075 *basB* transposon mutant, tnab1_kr130913p04q187	Gallagher et al. (2015) (39)
A. baumannii AB5075 *basE* transposon mutant, tnab1_kr130916p02q117	Gallagher et al. (2015) (39)
A. baumannii AB5075 *basD* transposon mutant, tnab1_kr130917p06q182	Gallagher et al. (2015) (39)
A. baumannii AB5075 *basD* transposon mutant, tnab1_kr130913p06q115	Gallagher et al. (2015) (39)
A. baumannii AB5075 *basD* transposon mutant, tnab1_kr130916p05q121	Gallagher et al. (2015) (39)
A. baumannii NY9	Bratu et al. (2008) (50)
A. baumannii NY30	Bratu et al. (2008) (50)
A. baumannii ACICU	Iacono et al. (2008) (51)
A. baumannii Clinical 1	Centre Hospitalier Universitaire de Caen
A. baumannii Clinical 2	Centre Hospitalier Universitaire de Caen
A. baumannii Clinical 3	Centre Hospitalier Universitaire de Caen
A. baumannii Clinical 4	Centre Hospitalier Universitaire de Caen
A. baumannii Clinical 5	Centre Hospitalier Universitaire de Caen
S. epidermidis 12228	ATCC
S. epidermidis 35984	ATCC
S. epidermidis 1	This study
S. epidermidis 2	This study
S. hominis	This study
*S. haemolyticus*	This study
C. striatum 6940	ATCC
*C. propinquum* 51488	ATCC

**TABLE 3:** Primers and plasmids utilized

Name	Source	Primer sequence
A1S_2382-3 recombine F	This study	GACGGACAAGCTATATATTCACAGCAAAATTGGAAATGATTAACAAATGCAACTGGTAATCATTTTCATTTGTTTGTATGATGCTGAAACAAGATTAATTTGTCAACCGAGTTATCGTTCACCGGAATTGCCAGCTGGG
A1S_2382-3 recombine R	This study	GATGAGACAAAAGAGCAGCTAAACCTAAGTTGAATGGCCCAATACCAATTCCGGTAATATCTATTTTTTTCATGTTTATACTTTATCTGTTTCCAAAATGATGAAAGTTCAAAATACGAATTCAGAAGAACTCGTCAAG
A1S_2382-3 screen F	This study	CTTGGTAAATTTTCTTACATATCGGCATA
A1S_2382-3 screen R	This study	CTGGTTTACGTTCTAAAAATACGC
AB5075_basA_screen	This study	CGAATCTCCCACCTCAGCTACTGC
AB5075_basB_screen	This study	CTCCATCTGCTGCCGATTTAGCTC
AB5075_basD_screen	This study	CCGGTGAGAGCTGAATCGCGTATGTTTC
AB5075_basE_screen	This study	GGTATAAGGCCCCCGAGTCGC
AB5075_basG_screen	This study	CCATATGGTGTAAGGTTGCGATATCGTC
AB5075_bauB_screen	This study	CCTACAGGTCAGGTGACGTCACTC
TN 26 seq primer Pgro-172	Gallagher et al. (2015) (39)	TGAGCTTTTTAGCTCGACTAATCCAT
27F-HT	Tyson et al. (2004) (48)	AGRGTTTGATYMTGGCTCAG
1492R-HT	Tyson et al. (2004) (48)	GGYTACCTTGTTACGACTT

### Data availability.

All data are available upon request.
